# Analysis of fetal fraction in non-invasive prenatal testing with low-depth whole genome sequencing

**DOI:** 10.1016/j.heliyon.2024.e41563

**Published:** 2024-12-28

**Authors:** Xiaolei Xie, Weiguo Yin, Fuguang Li, Suxia Xuan, Yu Ouyang

**Affiliations:** Molecular Diagnosis Center, Affiliated Qingyuan Hospital, Guangzhou Medical University (Qingyuan People's Hospital), 511518, Qingyuan, China

**Keywords:** Cell-free DNA, Fetal fraction, NIPT, Maternal weight, Trisomy 21

## Abstract

**Background:**

The fetal fraction (FF) is a critical factor influencing the performance of non-invasive prenatal testing (NIPT). Different NIPT methods and sequencing depths can lead to distinct minimum FF thresholds for Trisomy 21 (T21). This study aims to analyze the minimum FF thresholds for detecting T21 in PCR-free NIPT using a low-depth whole genome sequencing method.

**Methods:**

A total of 71 non-pregnant women and 4827 pregnant women were enrolled for a study investigating the impact of FF on NIPT testing.

**Results:**

The results showed a weak negative correlation between cfDNA levels and age in non-pregnant women. There was no statistically significant difference in the ratio of chromosome 13, 18, and 21 between pregnant and non-pregnant women. The minimum FF required for 100 % detection of trisomy 21 was determined to be 5 % in PCR-free NIPT using a low-depth whole genome sequencing method. Analysis of a large sample revealed that 4.43 % of pregnant women exhibited FF below 5 %, with those having lower FF showing significantly higher maternal weight compared to those near the median. Furthermore, FF demonstrated a significant negative correlation with the weight of pregnant women; specifically, an FF of 5 % at 12–19 weeks corresponded to a weight of 96 kg, while at 20–22 weeks it corresponded to a weight of 93 kg.

**Conclusions:**

These results suggest that the FF below 5 %, or pregnant women weighing over 93 kg, should be given more attention in clinical genetic counseling.

## Highlights

• Maternal cfDNA has little impact on NIPT.
• The minimum fetal fraction required for detection of trisomy 21 was 5 %.
• The prevalence of FF<5 % in pregnant women was 4.43 %, and those with higher maternal weight.
• The FF below 5 %, or pregnant women weighing over 93 kg, should be given more attention in clinical genetic counseling.

## Graphical abstract

**Figure:**
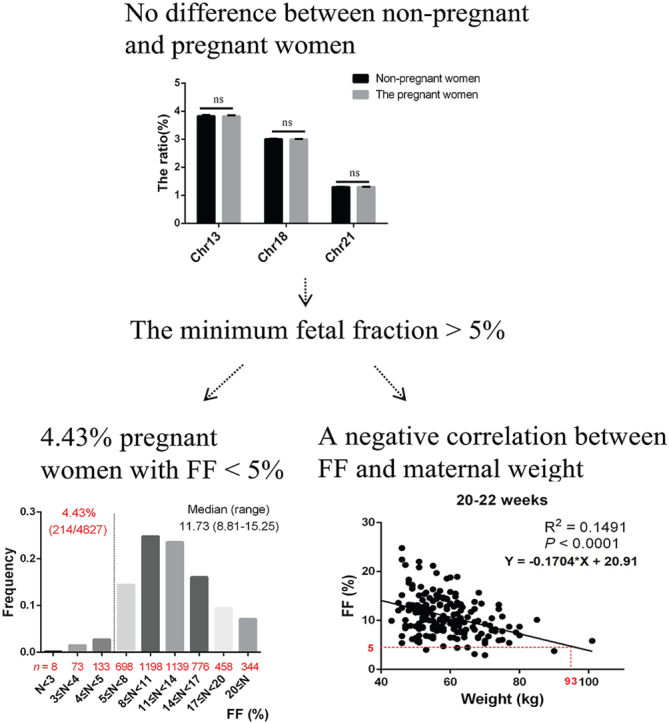

## 1 Introduction

The non-invasive prenatal testing (NIPT) technology was developed based on the discovery of cell-free fetal DNA (cffDNA) in 1997 [1,2], which utilized high-throughput gene sequencing to assess the risk of common fetal chromosome aneuploidies by detecting cell-free DNA (cfDNA) in maternal peripheral blood [3]. NIPT has demonstrated high sensitivity and specificity in detecting common trisomies (T21, T18 and T13) and sex chromosome abnormalities [4,5]. A false negative result for T21 may lead to the birth of children with Down syndrome. Overcoming false negatives for T21 poses a significant challenge in current NIPT detection. Various factors, including low fetal fraction [6,7], placental mosaicism [8,9], and maternal copy number variation (CNV) [10], can result in a false negative for T21.

Fetal Fraction (FF) was one of the key factors influencing NIPT performance [11]. A low fetal fraction may lead to the masking of a chromosomal abnormality by the predominant proportion of euploid maternal cfDNA, potentially resulting in false negative results in NIPT [6,7]. Relevant literature and the NIPT standard operating procedure (SOP) currently recommend a minimum fetal fraction threshold of 3.5 %, indicating that FF greater than 3.5 % is capable of detecting Trisomy 21 [12]. Additionally, a cut-off FF for T21 has been set at 4 %, determined by employing a statistical model based on sufficient sequencing read depth [9,13]. Furthermore, it has been noted that using the massively parallel sequencing (MPS)-NIPT method sets the limit of detection for T21 at the 2 % FF level [14]. Different methods and depths of sequencing can lead to distinct minimum FF thresholds for T21 detection. Currently, the minimum fetal fraction thresholds for T21 detection in PCR-free NIPT with low-depth whole-genome sequencing are still limited.

This study initially investigated the influencing factors of cfDNA in non-pregnant women and compared the characteristics and chromosome ratio of cfDNA between non-pregnant and pregnant women. Subsequently, it aimed to establish minimum fetal fraction thresholds for T21 by utilizing a model system to simulate samples with varying FFs, and to analyze the impact of maternal weight on the FF at different gestational ages.

## 2 Material and methods

### 2.1 Design and clinical data

Firstly, we recruited 71 non-pregnant women from whom we obtained parameters including height, weight, and age, additionally drawing 5 mL of peripheral blood for analysis purposes. Secondly, a retrospective study was conducted at the Affiliated Qingyuan Hospital of Guangzhou Medical University. A total of 4827 pregnant women undergoing PCR-free NIPT testing were enrolled. The time frame included September 2019 to December 2019 and January 2021 to May 2022. Pregnant women with intermediate (cut-off at 1/1000 and 1/270) or high-risk (cut-off at 1/270) serum screening, or advanced age (≥35 years) were recommended for NIPT testing. Less than 12 weeks of gestation was not recommended. Prenatal diagnosis was performed for pregnant women with high-risk NIPT results on trisomy 21 (T21), trisomy 18 (T18), trisomy 13 (T13), sex chromosomal abnormalities (SCA), and rare autosomal abnormalities (RAAs). Women with low-risk NIPT results were followed up until fetal birth. The control women were those of reproductive age who were not pregnant. All patients signed the informed consent form. The study was approved by the ethics committee of the Affiliated Qingyuan Hospital of Guangzhou Medical University (No. IRB-2022-064, June 21, 2022; No. IRB-2023-144, November 1, 2023 and No. IRB-2019-018, November 14, 2019).

### 2.2 Body fat rate and basal metabolism measurement

The portable upper-body type device (Omron HBF-306) was used to measure the body fat rate and basal metabolism of non-pregnant women. The parameters of gender, height, and weight were inputted into the device, and both hands were positioned horizontally within the sensing area for measurement.

### 2.3 CfDNA fragment analysis

Ten milliliters of blood were collected from non-pregnant and pregnant women using a cell-free DNA BCT™ tube (Streck, Omaha, USA). The blood was centrifuged first at 1600 g for 10 min at 4 °C. The plasma was then transferred to microcentrifuge tubes and further centrifuged at 16,000 g for 10 min.

Total cfDNA was extracted from 200 μL of plasma samples using a cfDNA extraction kit (BGI Company, Shenzhen, China) for subsequent analysis of cfDNA fragments. The process of cfDNA fragment analysis involved the preparation of a cfDNA library followed by polymerase chain reaction (PCR). CfDNA was subjected to end-repair to form blunt-ended DNA, followed by adaptor ligation. The purified cfDNA underwent PCR amplification on a Veriti™ 96 PCR machine (Applied Biosystems, USA). The PCR protocol was as follows: 2 min at 98 °C, 17 cycles of 98 °C for 15s, 56 °C for 15s, 72 °C for 30s and 5 min at 72 °C. The PCR products were subjected to fragment analysis using the Qsep100 machine (Bioptic Inc., China).

### 2.4 Artificial mixture preparation

To determine the limit of detection for the low-depth whole-genome sequencing NIPT method, we developed an experimental model that closely mimicked real clinical samples. This involved creating artificial mixtures with varying proportions of plasma samples from T21 male fetuses with known FF and samples from euploid female fetuses in pregnant women.

Expected FF (%) = C_1_∗V_1_∗FF%/(C_1_∗V_1_+C_2_∗V_2_)

C_1_ represents the library concentration of T21 male fetuses; V_1_ represents the library volume of T21 male fetuses; FF% represents the fetal fraction of T21 male fetuses; C_2_ represents the library concentration of euploid female fetuses, which share the same Barcode as C1; and V_2_ represents the library volume of euploid female fetuses.

The resulting artificial mixture cfDNA library was sequenced following Illumina NextSeq CN500 instructions. The expected FF was calculated based on the Y chromosome ratio after sequencing [15]. All samples in the artificial mixture exhibited a chrY z value of >15. The FF calculation of the PCR-free NIPT for female fetuses was based on the model calculation methods of Berry Genomics Company (Berry Genomics Company, Beijing, China).

### 2.5 The PCR-free NIPT

Ten milliliters of blood were collected from pregnant women. The blood was centrifuged first at 1600 g for 10 min at 4 °C and further centrifuged at 16,000 g for 10 min to remove residual cells. The cfDNA in 1.2 ml of blood plasma was extracted using the Berry Genomics Nucleic Acid Kit (Berry Genomics Company, Beijing, China). All NIPT tests did not include the PCR process. Total cfDNA was subjected to end repair to form blunt ends DNA, and then to adaptor ligation. A total of 95 DNA libraries were pooled equally. The library concentration of each sample needed to be greater than 10 pM before sequencing. The pooled sample library was loaded into a NextSeq flow cell at a DNA concentration of 3 pM. Clustering and sequencing were conducted according to Illumina NextSeq CN500 instructions using the single-ended 45 bp sequencing protocol. Unique Reads ≥1.5 Mb per sample were subjected to bioinformatics analysis. The z value of chr N equal to or greater than 3 indicates a high risk of chr N trisomy.

The sequencing reads were analyzed using the BambniTest software (Berry Genomics Company, Beijing, China). Unique reads that mapped exclusively to a single chromosome were selected for analysis. The assessment of fetal aneuploidy risk was conducted according to the z-test. Data from 412 pregnant women with euploid fetuses were defined as the reference set. The FF was below 3.5 %, and the software system indicated a low FF alert. Samples were then recommended for blood re-collection or abandon testing.

### 2.6 Statistical analysis

Data were analyzed using Graphpad Prism 6.0 software. Regression analysis (Pearson's correlation) was used to assess the relationship between cfDNA concentration and women's parameters. Multiple groups were analyzed using one-way ANOVA, while two groups were analyzed using an unpaired *t*-test. The values of maternal characteristics were presented as the mean (interquartile range). Statistical significance was set at ∗*P* < 0.05 and ∗∗∗∗*P* < 0.0001.

## 3 Results

### 3.1 Comparative analysis of cfDNA between non-pregnant and pregnant women

**Fig. 1:**
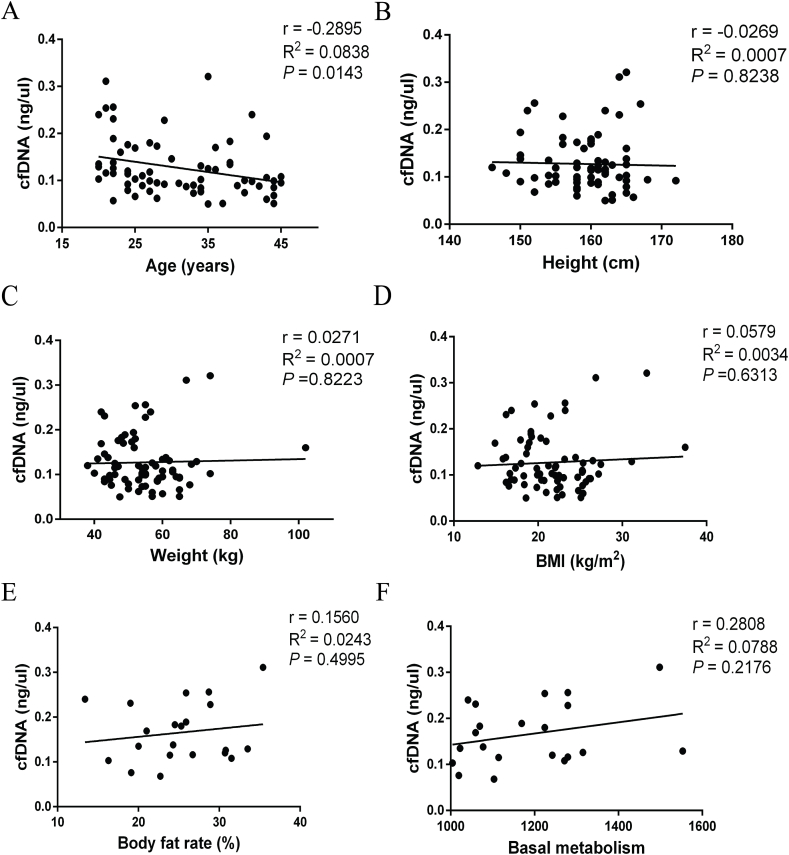
The analysis of factors influencing the levels of cfDNA in non-pregnant women. The levels of cfDNA exhibit a weak negative correlation with women's age (A), while no significant correlations were observed with height (B), weight (C), BMI (D), body fat rate (E), or basal metabolism (F).

To investigate the factors influencing cfDNA levels in non-pregnant women, we previously recruited 71 non-pregnant women for analysis of these influencing factors. The age range of participants was from 20 to 45 years, with a mean age of 30.79 years and a mean weight of 54.76 kg. Our findings suggest a weak negative correlation between cfDNA levels and women's age (Fig. 1A), while no significant correlations were observed with height (Fig. 1B), weight (Fig. 1C), BMI (Fig. 1D), body fat rate (Fig. 1E), or basal metabolism (Fig. 1F).

**Fig. 2:**
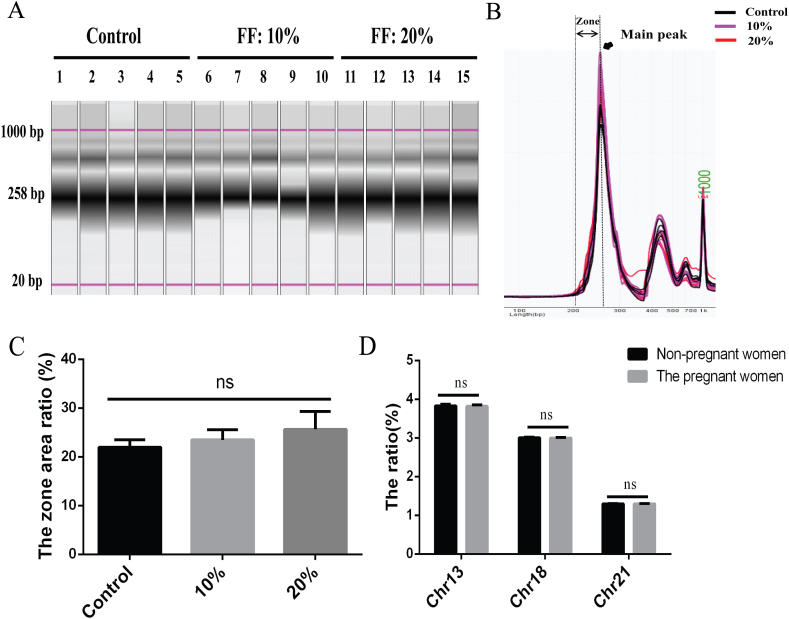
Comparison of cfDNA characteristics between non-pregnant and pregnant women. (A) Gel analysis of cfDNA in non-pregnant and pregnant women. (B) Fragment analysis of cfDNA at different FFs. (C) The zone area ratio gradually increased from control to 20 % FF, but no significant difference was observed. Control: non-pregnant women; 10 % and 20 %: levels of cffDNA at 10 % and 20 % in pregnant women. n = 5. (D) The ratio of chromosomes 13, 18, and 21 showed no significant difference between non-pregnant and pregnant women. Error bars represent SD, One-way ANOVA for the zone area ratio, Unpaired *t*-test for chromosome ratios.

To compare the characteristics of cfDNA, we further selected 5 samples from each cohort for fragment analysis. CffDNA fragments exhibit a main peak at 143 bp, which is shorter than that of cfDNA fragments with a main peak at 166 bp. The results indicated a gradual increase in the zone area ratio from the control to 20 % FF, with the zone area corresponding to the region where cffDNA's main peak appeared (Fig. 2A–C). Subsequently, forty-five non-pregnant women were chosen for next-generation sequencing, and concurrently, data from 100 cases of NIPT detection were randomly selected for comparison. The ratios of chromosomes 13, 18, and 21 showed no significant differences between non-pregnant and pregnant women (Fig. 2D). These findings suggest that maternal cfDNA has a limited impact on NIPT detection.

### 3.2 The minimum detectable limit of trisomy 21

**Fig. 3:**
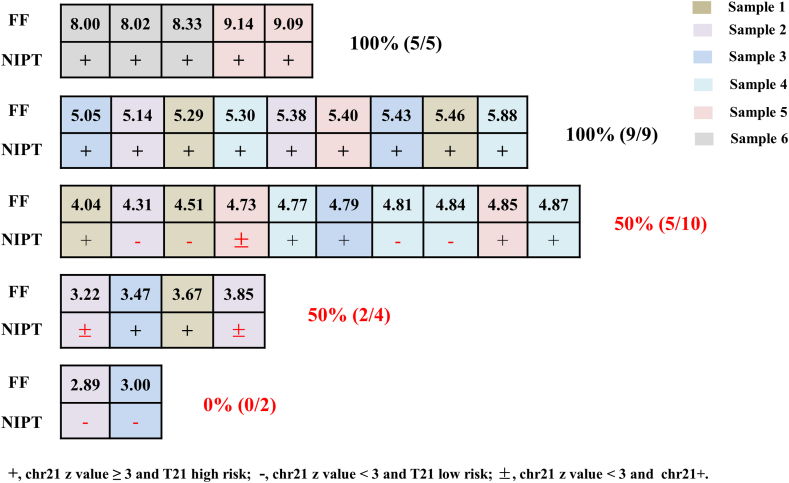
Results of NIPT testing with different fetal fractions of Trisomy 21.

We further selected 6 samples of male fetuses with a positive NIPT test for trisomy 21, and these samples were subsequently confirmed as true positives by prenatal diagnosis. In vitro, six samples of mixed female fetuses samples were diluted to achieve a range of FF concentrations: <3 %, 3 % < FF < 4 %, 4 % < FF < 5 %, 5 % < FF < 6 %, and FF > 8 %.The results indicated that a positive detection rate of 100 % can be achieved when FF > 5 %, while the positive detection rate for the range 4 % < FF < 5 % is 50 % (Fig. 3).

We subsequently conducted a retrospective analysis of 4827 NIPT results. Maternal characteristics were presented in Table 1. The results of T21 were further analyzed, and the minimum FF in true positive T21 was 4.88 % (Table 2).

**Table 1:** The characteristics of pregnant women groups.

	Pregnant women
No. of individuals	4827
Mean maternal age (range)-yr	30.92 (27–35)
Mean gestational age (range)-wk	16.48 (14–18)
Mean maternal weight (range)-kg	56.43 (50–62)
Twin rate (%)	1.20 % (58/4827)
Advanced maternal age (≥35)	28.67 % (1384/4827)
NIPT results	
T21 (TP + FP)	16 (14 + 2)
T18 (TP + FP)	4 (1 + 3)
T13	0
SCA(TP + FP)	12 (8 + 4)
RAAs (TP + FP)	9 (0 + 9)

TP:True positive; FP: False positive; SCA: Sex chromosomal abnormalities; RAAs: Rare autosomal abnormalities.

**Table 2:** Analysis of the detection results of T21.

	Age (years)	Gestational age (weeks)	The z value of Chr21	FF (%)
True positive				
1	36	16	10.81	13.91
2	38	17^+4^	13.40	14.43
3	40	25^+4^	24.83	28.03
4	47	14	10.46	16.07
5	38	14^+5^	13.35	20.06
6	46	21^+3^	7.85	11.34
7	41	13^+4^	3.83	4.88
8	36	15^+4^	9.77	12.55
9	27	17^+5^	6.43	12.73
10	29	12^+3^	11.64	16.20
11	28	13^+5^	13.51	15.83
12	41	13^+6^	6.79	12.22
13	23	15^+6^	12.44	14.80
14	40	18^+4^	9.90	14.38
False positive				
1	26	31	3.81	13.91
2	30	18^+1^	3.04	24.9
False negative				
1	35	17^+3^	1.78	8.13

### 3.3 Analysis of FF in pregnant women

**Fig. 4:**
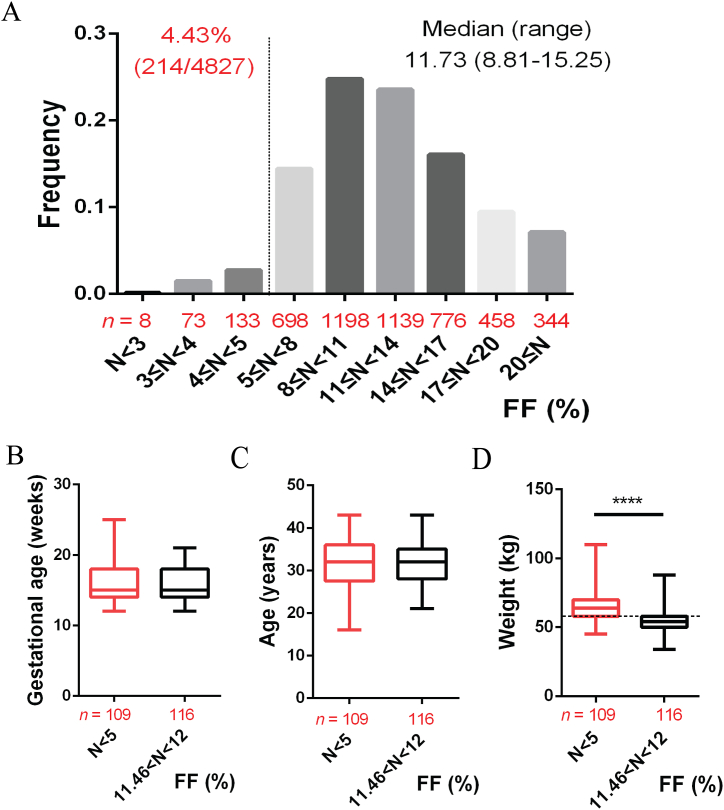
Analysis of the distribution frequency of FF in pregnant women (A), as well as comparisons of gestational age (B), maternal age (C), and maternal weight (D) between FF below 5 % and near the median FF. ∗∗∗∗*P* < 0.0001, Unpaired *t*-test.

Retrospective clinical data reveal that the median fetal fraction (FF) was 11.73 %, with FF values below 5 % accounting for 4.43 % (214/4827) (Fig. 4A). Further analysis of the sample data between the FF < 5 % group and the group near the median revealed that there were no significant differences in gestational age or maternal age between the two groups (Fig. 4B and C). However, maternal weight was found to be significantly higher in the FF < 5 % group compared to the group near the median (Fig. 4D).

**Fig. 5:**
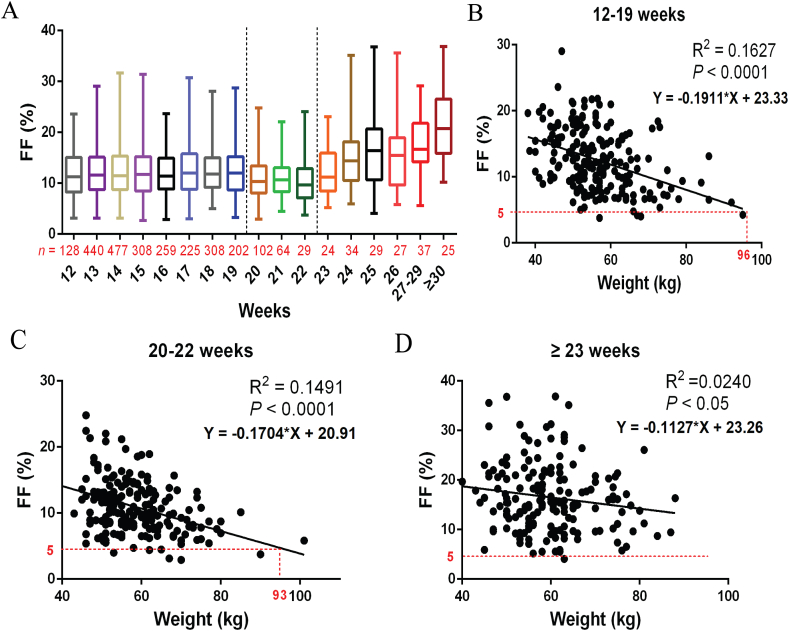
The level of FF in different gestational age (A). The correlation between maternal weight and FF was analyzed in 12–19 weeks (B), 20–22 weeks (C), and ≥23 weeks (D).

Maternal weight and gestational age were two distinct factors that significantly impacted FF. Our data confirm the observation that FF gradually increases with gestational age (Fig. 5A). However, FF remained relatively constant in weeks 12–19, decreased slightly in weeks 20–22, and increased rapidly from weeks 23. There was a significant negative correlation between FF and maternal weight in different gestational ages, among which the weight corresponding to 5 % FF at 12–19 weeks was 96 kg (Fig. 5B), at 20–22 weeks was 93 kg (Fig. 5C), and at ≥23 weeks was 162 kg (Fig. 5D).

## 4 Discussion

The presence of a low fetal fraction in maternal plasma may lead to a false-negative T21 NIPT result. Analysis of cell-free DNA levels and sequencing in non-pregnant women has suggested that maternal cfDNA has little impact on NIPT. The low proportion of trisomy T21 indicated that the FF with 100 % detection was 5 %. A large sample study revealed that 4.43 % of pregnant women exhibited FF below 5 %, and the maternal weight of pregnant women with FF below 5 % was significantly higher. FF showed a significant negative correlation with the weight of pregnant women at different gestational ages. These results suggest that an FF below 5 %, or a pregnant woman's weight greater than 93 kg, should be given more consideration in clinical genetic counseling.

The distribution of cfDNA differs between the fetal and maternal genomes, enabling accurate quantification of the fetal fraction based on this distinction [16]. The majority of cfDNA from the mother may mask a small proportion of FF anomalies, leading to inaccurate NIPT results [6,7]. Therefore, we observed a modest negative correlation between cfDNA levels and age by recruiting non-pregnant women. Furthermore, there was no statistically significant difference in the ratio of chromosome 13, 18, and 21 between pregnant and non-pregnant women. These findings suggest that the influence of maternal factors on NIPT is minimal.

Different NIPT methods and sequencing depths may lead to varying minimum fetal fraction thresholds for T21. Literature reported that the minimum FF for detectable T21 was 2%–4% [13,14]. A low fetal fraction can be partially compensated by a higher sequencing depth, which helps reduce statistical noise. In this study, the minimum FF required for 100 % detection of trisomy 21 was determined to be 5 %. Approximately 6 bp of sample barcodes were eliminated during the bioinformatics analysis. Considering single-end reads of 45 bp sequencing, this procedure led to an effective genome sequencing length of 39 bp per read. Combined with the fact that Unique Reads were no less than 1.5 Mb per sample, consequently, the sequencing depth was calculated to be 0.0195 (1.5M ∗ 39 bp/3 Gb).

In clinical data, the minimum FF was 4.88 % in T21 true positive cases, suggesting that an FF greater than this ratio can effectively detect T21. Additionally, two cases of false positive T21 and one case of false negative T21 were identified. A variety of factors, such as confined placental mosaicism (CPM) or maternal CNV, might lead to false positive or false negative results of T21. In clinical practice, pregnant women with false positive or false negative T21 results were frequently reluctant to undergo further analytical testing.

Collecting more cases of T21 positive and T21 false negative in NIPT could potentially be beneficial for studying the detection limits of T21. In this study, we utilized T21 true positive cases to simulate various FFs to explore the influence of the PCR-free NIPT method on T21 detection. The study offers a reference for laboratory technicians and clinicians to evaluate the NIPT result, although in some cases the FF value does not appear in the report.

The minimum FF threshold of this PCR-free method was suggested to be 3.5 % in accordance with the standard operating procedure (SOP). If the fetal fraction in a case was lower than 3.5 %, it was necessary to recollect the blood. In this study, the FF with 100 % detection for T21 was 5 %. When the FF in a case was within the range between 3.5 % and 5 %, more attention should be given. To avoid T21 false negatives, it was recommended to recollect blood or reconstitute the DNA library for NIPT retesting, especially when fetal ultrasound abnormalities were identified. Because the FF during the blood recollecting period might increase, or reconstructing the DNA library for retesting could enhance the probability of T21 detection.

Previous studies have reported that gestational age and maternal weight are significant factors influencing FF [15,17]. Our results support a gradual increase in FF with the increase of gestational age. We observed a gradual increase in FF from 12 to 19 weeks, followed by a slight decline from 20 to 22 weeks, and then a significant rise at 23 weeks. Maternal weight exhibited a negative correlation with FF across different stages of gestational ages, consistent with our data and other research studies [11,15,17]. The minimum detectable limit of 5 % FF at 12–19 weeks was 96 kg, and 20–22 weeks was 93 kg, indicating that maternal weight greater than 93 kg should be paid more attention to FF during genetic counseling.

The study may have the following limitations: (1) Due to the limited sample size, we have selected only 6 true positive T21 samples for the detection limit experiment. Therefore, further investigation with a larger sample size is essential to validate our findings. (2) Different NIPT detection methods and sequencing depths can result in varying detection limits. Medical laboratories should establish their own minimum detection limits based on their NIPT detection methods to prevent missed T21 diagnoses. (3) In a specific NIPT test method, it is beneficial to collect more true positive and false negative T21 cases for the determination of the T21 detection limit. However, such data were limited in this study.

In summary, this study has demonstrated that maternal cfDNA has a minimal impact on NIPT. The minimum detection limit for T21 is 5 %, and the FF of maternal weight exceeding 93 kg is likely to be less than 5 %. The findings of this study offer valuable insights for genetic counseling in PCR-free NIPT using low-depth whole genome sequencing.

## CRediT authorship contribution statement

**Xiaolei Xie:** Writing – original draft, Conceptualization. **Weiguo Yin:** Writing – review & editing. **Fuguang Li:** Supervision. **Suxia Xuan:** Methodology, Data curation. **Yu Ouyang:** Methodology, Data curation.

## Ethics statement

This study was reviewed and approved by the ethics committee of the Affiliated Qingyuan Hospital of Guangzhou Medical University with the approval number: [No. IRB-2022-064], dated [June 21, 2022]; [No. IRB-2023-144], dated [November 1, 2023] and [No. IRB-2019-018], dated [November 14, 2019]. All participants provided written informed consent to participate in the study and for their data to be published.

## Data availability statement

The data that support the findings of this study are available on request from the corresponding author.

## Funding statement

This work was supported by the 10.13039/501100021171Guangdong Basic and Applied Basic Research Foundation [2021A1515220118], and the 10.13039/501100003785Guangdong Medical Research Foundation [A2024549 and A2020553].

## Declaration of competing interest

The authors declare that they have no known competing financial interests or personal relationships that could have appeared to influence the work reported in this paper.
